# Paramagnetic Structures within a Microfluidic Channel for Enhanced Immunomagnetic Isolation and Surface Patterning of Cells

**DOI:** 10.1038/srep29407

**Published:** 2016-07-08

**Authors:** Chen Sun, Hamid Hassanisaber, Richard Yu, Sai Ma, Scott S. Verbridge, Chang Lu

**Affiliations:** 1 Department of Biomedical Engineering and Mechanics, Virginia Tech, Blacksburg, Virginia 24061, USA; 2 Department of Chemical Engineering, Virginia Tech, Blacksburg, Virginia 24061, USA; 3 Department of Biomedical Engineering, Washington University in St. Louis, St. Louis, Missouri 63130, USA

## Abstract

In this report, we demonstrate a unique method for embedding magnetic structures inside a microfluidic channel for cell isolation. We used a molding process to fabricate these structures out of a ferrofluid of cobalt ferrite nanoparticles. We show that the embedded magnetic structures significantly increased the magnetic field in the channel, resulting in up to 4-fold enhancement in immunomagnetic capture as compared with a channel without these embedded magnetic structures. We also studied the spatial distribution of trapped cells both experimentally and computationally. We determined that the surface pattern of these trapped cells was determined by both location of the magnet and layout of the in-channel magnetic structures. Our magnetic structure embedded microfluidic device achieved over 90% capture efficiency at a flow velocity of 4 mm/s, a speed that was roughly two orders of magnitude faster than previous microfluidic systems used for a similar purpose. We envision that our technology will provide a powerful tool for detection and enrichment of rare cells from biological samples.

Microfluidics provides an effective platform for manipulating small quantities of cell samples with sensitivity and precision^1^,^2^,^3^. Isolation and enrichment of a specific cell type from complex biological samples are critical for molecular studies and clinical applications. With microscale channels and chambers, microfluidic devices are ideal for isolating samples in the 10–10,000 cell range^1^,^4^,^5^,^6^. Various microfluidic isolation technologies have been developed over the years, targeting the biophysical properties and surface biomarkers of cells^1^,^7^,^8^. Physical methods separate and isolate cells based on their size and density^9^,^10^,^11^, deformability^12^,^13^, electrical polarizability^14^,^15^, and intrinsic magnetic susceptibility^16^. Despite their success, most physical methods do not involve identification and selection based on biomarkers, thus may lack specificity or biological utility especially when used on highly heterogeneous cell populations such as primary tissues. In comparison, immunoassay-based isolation methods isolate cells by targeting unique surface markers via antibody-antigen interactions and offer high specificity that is critical for molecular biology studies^17^,^18^,^19^,^20^. Among various immunoassay-based isolation methods, immunomagnetic separation (IMS) is implemented by using a magnetic field to manipulate magnetically labeled cells, and this method is gaining popularity due to its minimally invasive nature. Microfluidic devices working in both trapping mode (i.e. using a magnetic field to trap labeled cells inside channels/chambers^21^,^22^,^23^,^24^,^25^) and continuous sorting mode (i.e. using a magnetic field to direct labeled cells to desired outlets^26^,^27^,^28^,^29^) have been demonstrated.

The vast majority of the microfluidic IMS devices have applied a magnetic field via placing a magnet exterior to the microfluidic structures. However, because the magnetic field intensity decays rapidly with distance, it would be highly advantageous to place additional magnetic structures inside the microfluidic channels. This would furthermore provide a robust means of creating consistent field distributions in microfluidic devices. Previous work has involved complex microfabrication procedures (e.g. electroplating or sputtering) for fabricating microscale magnetic structures inside the microfluidic channels^26^,^27^,^30^,^31^,^32^,^33^,^34^. In order to generate a substantial thickness (~micrometers) for the magnetic structures for significant field enhancement, these procedures easily become costly and time-consuming and are not suitable for low-cost devices. Fabrication by molding PDMS structures that contained iron microparticles was demonstrated recently^25^,^29^.

In this report, we demonstrate a simple microfluidic device that contains microscale paramagnetic structures inside a microfluidic channel for cell isolation based on IMS. We used a molding process to fabricate these magnetic structures (with a thickness of 4.5 *μ*m) out of a ferrofluid of cobalt ferrite nanoparticles (5–13 nm in diameter). We tested our microfluidic channels for capturing RAW 264.7 cells that were magnetically labeled by microscale beads. We found that channels embedded with the magnetic structures yielded up to 4 times higher capture efficiency compared to that of channels without the structures. We built COMSOL models to simulate the magnetic field and magnetic field gradient distribution inside channels and generate insights into the mechanistic details. Both the experimental and modeling results indicated that the layout of the magnetic structures and the location of the magnet strongly affected the distribution of the trapped cells. With the enhanced magnetic field gradient, our device allowed 100% capture efficiency at a flow velocity of 0.5 mm/s and >90% capture rate at 4 mm/s. These velocities were 20–80 times faster than those of previous microfluidic IMS devices^7^,^21^,^22^,^23^,^35^.

## Results and Discussion

### Fabrication and characterization of embedded magnetic structures

**Figure 1:**
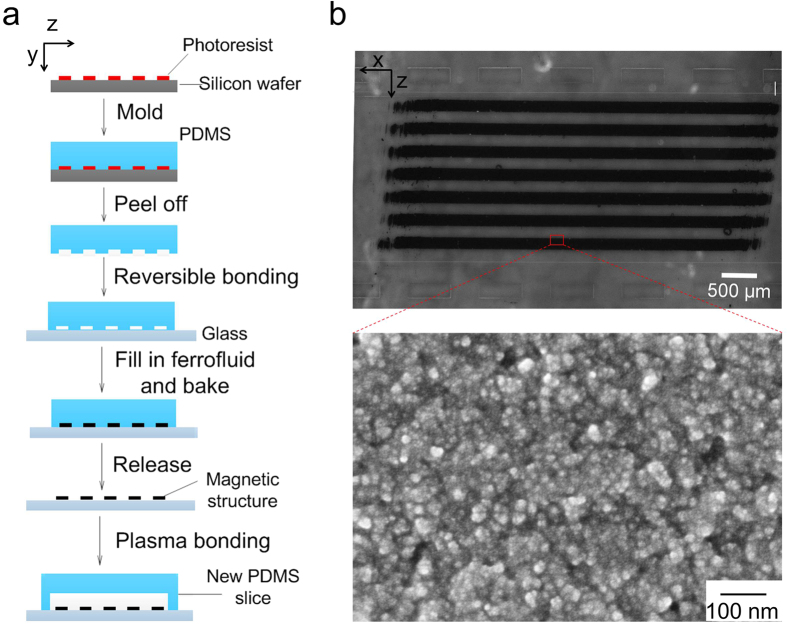
The fabrication and characterization of microfluidic magnetic structures. (**a**) The procedure of fabricating thick paramagnetic stripes via microfluidic molding of ferrofluidic ink. (**b**) Optical image of the magnetic stripes in a microfluidic channel. The dimensions of each stripe are 160 *μ*m (Width, z direction) × 4.5 *μ*m (Depth, y direction) ×5 mm (Length, x direction) with a gap of 160 *μ*m in between. The magnetic structure was located within a PDMS microfluidic channel that was 2.4 mm wide and 100 *μ*m deep. Scale bar: 500 *μ*m. The inset shows a SEM image of the magnetic structure. Scale bar: 100 nm.

Magnetic structures were fabricated by microfluidic molding. As shown in Fig. 1a, a microfluidic network with desired geometry and dimensions was first fabricated by soft lithography into polydimethylsiloxane (PDMS). The PDMS microfluidic structure (which served as a mold) was put into contact with a clean glass substrate to form a reversible seal. Ferrite ink (i.e. cobalt ferrite nanoparticles in water) was filled into the microfluidic network and then the entire structure was baked for 15 min intervals at increasing temperatures of 30, 40, 50 and 60 °C. The PDMS structure was then peeled off the glass substrate carefully before an additional 10–12 h heating step at 70 °C was conducted. The ferrite ink left solid magnetic structures defined by the PDMS mold, after evaporation of water by baking. With the PDMS mold having a channel depth of 10 *μ*m, the thickness of the magnetic structure was ~4.5 *μ*m (Fig. 1b). A new PDMS microfluidic channel was then aligned with the magnetic structures on the glass substrate and irreversibly bonded to the glass substrate after plasma treatment to form a closed channel. The inset scanning electron microscope (SEM) image shows that our magnetic structure was assembled by cobalt ferrite nanoparticles with diameters smaller than 10 nm with a high density. The attachment of the magnetic structures to the glass substrate was very robust and could sustain a flow up to 10 cm/s. There was no visible loss of the magnetic material under prolonged duration of flow (up to 3–4 h).

### Immunomagnetic labeling and capture of CD11b^+^ cells

**Figure 2:**
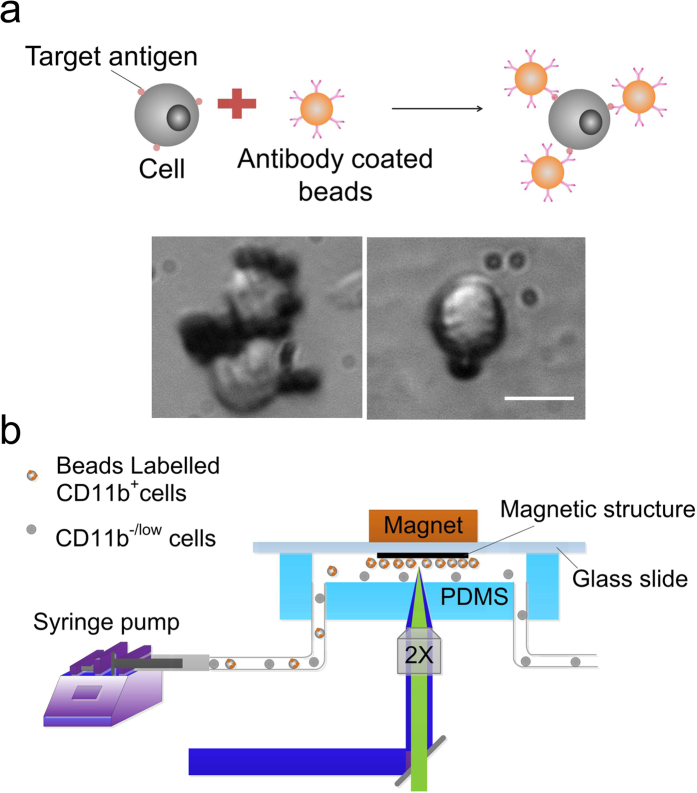
Labeling of CD11b^+^ cells by magnetic beads and capture of them in a microfluidic channel. (**a**) Magnetic labeling of cells via immunoassay. Antibody-coated magnetic beads bind to target cells. The inset shows images of bead-bound RAW 264.7 cells. Scale bar: 10 *μ*m. (**b**) The experimental setup for our microfluidic immunomagnetic separation. A NdFeB magnet was placed on the glass slide side of the microfluidic device to reduce the distance between the magnet and the channel.

RAW 264.7 cells (a mouse leukaemic macrophage cell line) were conjugated with functionalized magnetic beads (Dynabeads, 4.5 *μ*m in diameter, Life technologies) via antigen-antibody interaction, as shown in Fig. 2a. The surface of the magnetic beads was functionalized with streptavidin via a DNA linker and then linked with a biotinylated antibody via streptavidin-biotin interaction, to bind leukocyte-expressed CD11b. These beads can be released from the cell surface by cleaving the linkage between beads and streptavidin using DNase if further analysis of cells is needed. 89% of the cells were labeled by the magnetic beads after mixing the cells (examined by optical imaging) and beads at a ratio of 1:4 for 1 h at 4 °C under rotation. This percentage matches the percentage of CD11b-expressing cells in RAW 264.7 reported in the literature^36^. The experimental setup is shown in Fig. 2b. A permanent NdFeB magnet (1/4″ × 1/4″ × 1/4″) was placed on the glass side in order to achieve a small distance (the thickness of the glass slide was 1 mm) between the magnet and the channel. Biocompatible high molecular-weight dextran was added in the buffer to match the liquid and cell densities to avoid settling^37^,^38^,^39^. We added 7%wt of dextran in phosphate buffer saline (PBS) and found no substantial settling of cells at the entry of the channel in the experiments under the flow velocities specified (i.e. 4–20 mm/s). The cell/bead suspension was introduced into the channel at a constant flow rate sustained by a syringe pump. The availability of surface area may affect cell capture efficiency. In our experiments, lower than 50% of the channel surface was covered by cells at the end of these tests.

### Embedded magnetic structures enhance cell capture efficiency

**Figure 3:**
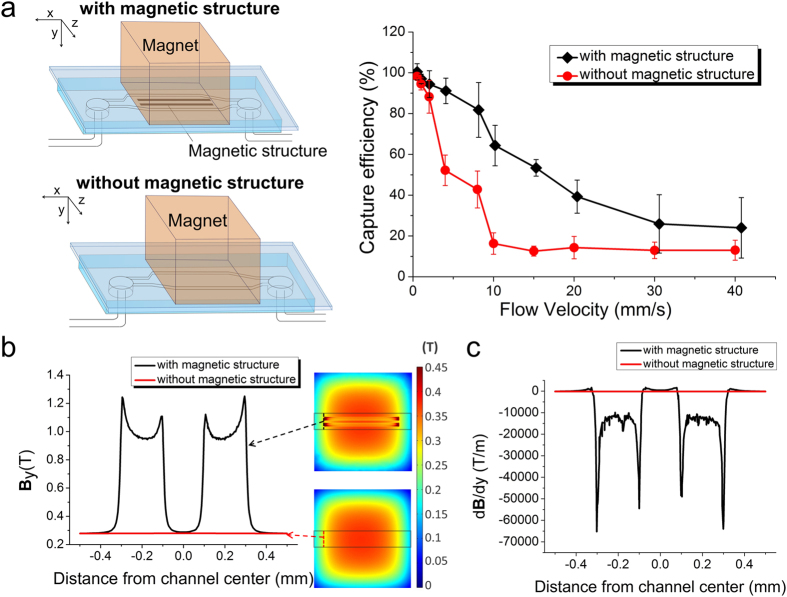
Paramagnetic structure increases cell capture efficiency in a microfluidic IMS device. (**a**) Cell capture rate (i.e. the percentage of captured cells among all magnetically labeled cells) as a function of flow rate in a microfluidic channel containing the magnetic structure (black diamonds) and that without the structure (red circles). (**b**) Magnetic flux density ***B***_*y*_ modeled by COMSOL Multiphysics. ***B***_*y*_ (in a plane that is within the channel and 10 *μ*m from the glass surface) with the magnetic structure (black) and the one without it (red) plotted against the distance from the channel center (along the channel width). (**c**) Magnetic field gradient *d**B***/*dy* modeled by COMSOL Multiphysics at the upstream end of the magnetic stripes. *d**B***/*dy* in the channel with the magnetic structure (black) and the one without it (red) plotted against the distance from the channel center (along the channel width). The residual flux density of the external magnet was set at 1.3 Tesla. The dimensions of the microfluidic channel were 0.96 mm (Width, z direction) × 100 *μ*m (Depth, y direction) × 8 mm (Length, x direction), and the magnetic structure included 2 magnetic stripes of 200 *μ*m (W) × 4.5 *μ*m (D) × 5 mm (L) with a gap of 200 *μ*m in between.

We optimized the flow conditions to facilitate trapping of magnetically labeled cells and removal of unlabeled cells. We studied cell capture efficiency in microfluidic channels with and without the embedded magnetic microstructures as a function of flow rate. In either case, the microfluidic channel had a width of 0.96 mm and a depth of 100 *μ*m. In the channel with the magnetic structures, magnetic stripes that were 200 *μ*m wide, 4.5 *μ*m thick and 5 mm long, separated by a 200 *μ*m gap, were fabricated on the glass substrate. The permanent NdFeB magnet was placed on the glass side to cover the entire magnetic stripe-filled area. Cells were fluorescently labeled by calcein-AM to facilitate imaging. The capture efficiency was defined as the number of magnetically labeled cells (accounting for 89% of the total cell population, as described above) trapped inside the channel divided by the total number of magnetically labeled cells that flow through the channel during the period. As shown in Fig. 3a, generally the capture efficiency decreased as the flow velocity increased in both channels (i.e. with and without the magnetic structures), due to the increased drag force associated with the higher velocity. The cell capture efficiency in the channel with the magnetic stripes was substantially higher compared with the channel without such features under medium velocities (4–20 mm/s). The difference diminished under very high/low velocities when the magnetic force was either too low/high compared to the drag force exerted by the flow. When the flow velocity was low, the capture efficiencies approached 100% under both conditions (i.e. with or without the embedded structure). The channel with the magnetic stripes yielded a capture efficiency ranging from 91% to 39% under flow velocities 4–20 mm/s, compared to 52% to 14% yielded by the channel without the structure. The enhancement was as high as 4-fold at a flow velocity of 10 mm/s (64% vs. 16%). It is worth noting that the flow velocity used in our studies (4–20 mm/s) was significantly faster than that of other microfluidic IMS assays reported by other groups (50–200 *μ*m/s)^22^,^35^,^40^. In all cases, the captured cells can be 100% eluted from the channel after removing the magnet. The fabricated structures are paramagnetic in nature and the magnetic field becomes negligible after the permanent magnet is removed.

The magnetic force ***F*** on a magnetic particle is governed by ***F*** = ***m∇B***, where ***m*** is the magnetic moment of the particle, ***B*** is the magnetic flux density^26^,^30^,^41^,^42^,^43^,^44^. The magnetic moment of the particle ***m*** = Δ*χV**B***/*μ*_0_, where Δ***χ*** is the difference in magnetic permeability between the particle and surrounding buffer, *V* is volume of the particle, and *μ*_0_ is the magnetic permeability constant. ***m*** increases with ***B*** and reaches a saturation value *m*_*s*_, with a direction that is parallel to ***B***. Under these circumstances, ***F*** = *m*_*s*_*∇**B*** and is proportional to ***∇B***^26^,^30^. The magnetic field and field gradient in the channels with or without magnetic stripes was modeled using COMSOL Multiphysics. We only considered ***B*** and ***∇B*** in the direction that is perpendicular to the glass substrate (***B***_***y***_ and *d**B***/*dy*) only, because the magnetic force in this direction provided the main mechanism for cell trapping. As shown in Fig. 3b,c, both ***B***_***y***_ and *d**B**/dy* were substantially enhanced in the space that was on top of the magnetic stripes, especially at the ends of the stripes. *d**B***/*dy* (and***F***) in this region was about 50 times stronger than that without the magnetic stripe underneath.

### Spatial distribution of trapped cells was affected by both the location of the magnet and the layout of the magnetic structures

**Figure 4:**
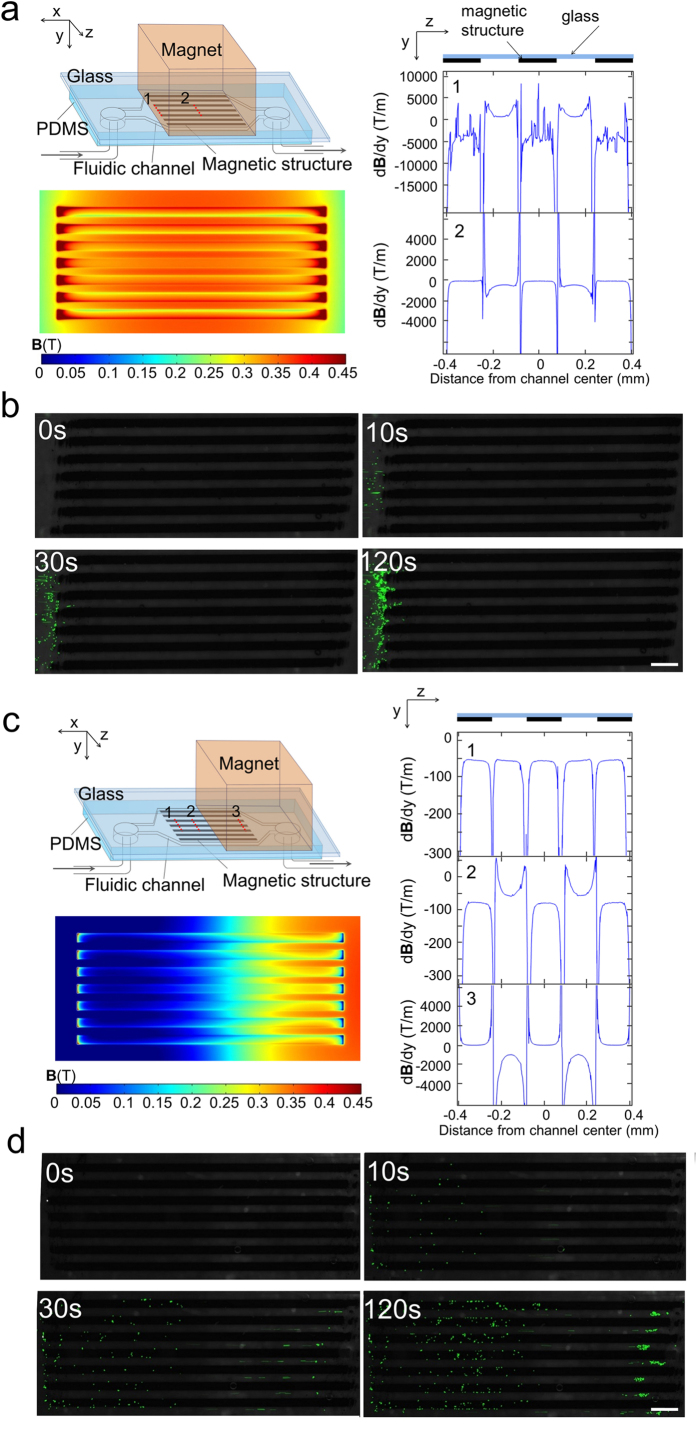
The layout of the magnetic structures and the location of the magnet determine the spatial distribution of trapped cells in a microfluidic channel. (**a**) COMSOL modeling of ***B***_*y*_ and *d**B**/dy* (in the plane that is within the channel and 10 *μ*m from the glass surface) when the magnet is placed right above, covering the magnetic structure. *d**B***/*dy* was plotted along the channel width at various locations (1 and 2) along the channel length. The fluctuation in the location 1 data was likely due to numerical artifact. (**b**) Time-lapse images of cell accumulation in the channel with the setting showed in (**a**). Scale bar: 500 *μ*m. (**c**) COMSOL modeling of ***B***_*y*_ and *d**B***/*dy* when the magnet covers only half of the magnetic structure. *d**B***/*dy* was plotted along the channel width at various locations (1, 2 and 3) along the channel length. (**d**) Time-lapse images of cell accumulation in the channel with the setting showed in (**c**). Scale bar: 500 *μ*m. The dimensions of the microfluidic channel were 2.4 mm (W, z direction) × 100 *μ*m (D, y direction) × 10 mm (L, x direction), and the magnetic structure contained 7 stripes of 160 *μ*m (W) × 4.5 *μ*m (D) × 5 mm (L) uniformly aligned in the channel. Cells were fluorescently labelled with calcein AM green.

Interestingly, trapped cells formed surface patterns that were dependent on both the location of the external magnet and the layout of the embedded structures. Fig. 4 shows two different locations of the magnet (in relation to the magnetic stripes) and the corresponding COMSOL models established to simulate the magnetic field ***B***_*y*_ and magnetic field gradient *d**B***/*dy.* When the magnet covers the entire magnetic structures from above, ***B***_*y*_ and *d**B**/dy* were the strongest at the upstream ends of the magnetic stripes (Fig. 4a). At location 1, *d**B***/*dy* on top of the magnetic stripes was around −4000 T/m (i.e. the magnetic force was strongly attractive) and the regions between the stripes showed *d**B***/*dy* value around 700 T/m (i.e. the magnetic force was repulsive). *d**B***/*dx* on top of the magnetic stripes exerted a magnetic force that is opposite to flow drag force in direction (Fig. S1a). As the net result, the time-lapse images in Fig. 4b (and SI Movie S1) show that magnetically labeled cells were trapped close to the upstream ends (location 1) of the magnetic stripes. In contrast, when the magnet covered only half of the magnetic stripes (i.e. with the magnet having its upstream edge at the middle of the stripe length) (Fig. 4c), COMSOL modeling indicated that *d**B***/*dy* was around −50 T/m at the upstream end of the magnetic stripes (location 1) across most of the channel width. *d**B**/dy* was strongly negative at the edges of the magnetic stripes. However, we believe that these extreme *d**B**/dy* intensities at the stripe edges do not substantially affect cell trapping due to the very narrow regions they influence. Then *d**B**/dy* became slightly higher on the stripes than between the stripes at the more downstream location 2 (with around −80 T/m on the magnetic stripes and −55 T/m between the stripes). *d**B***/*dx* was uniform across the channel width at both locations 1 and 2 (Fig. S1b). However, at location 3 (close to the downstream ends of the magnetic stripes), *d**B**/dy* was −1000 T/m between the stripes and 80 T/m on the magnetic stripes. *d**B**/dx* was around 1400 T/m on the magnetic strips and −100 T/m between the strips (Fig. S1b). Our modeling results explain what we observed experimentally (Fig. 4d and SI Movie S2). At location 1, we observed cell distribution that was essentially uniform throughout the channel surface. At location 2, cells showed obvious preference for occupying the space on the stripes and the edges of the stripes. However, in the region around location 3, cells occupied only the space between the stripes due to the strong attractive force there. As a comparison, we found that cells were stopped at random locations in the microfluidic channel without the magnetic stripes (SI Movie S3).

## Summary

To summarize, we have developed a simple molding method to fabricate paramagnetic structures with a thickness of several micrometers inside a microfluidic channel for enhanced immunomagnetic separation of cells. We show that the paramagnetic structures drastically increased the magnetic field and thus the capture efficiency of cells. The layout of the magnetic structures and the location of the external magnet collectively determine the spatial distribution of trapped cells, which may be leveraged for concentrating and patterning cells. Our technology will find applications for processing and analyzing rare cells from biological samples.

## Methods

### Cell culture and fluorescent labeling

RAW 264.7 cells (mouse leukemic monocyte macrophage cell; ATCC, TIB-71) were grown in Dulbecco’s Modified Eagle Medium (DMEM) (Life technologies) supplemented with 10% fetal bovine serum (FBS) (Atlanta Biologicals) and 1% penicillin/streptomycin (Invitrogen) in a humidified atmosphere of 5% CO_2_ at 37 °C. Cells were subcultured every 2 days at a ratio of 1:10 to maintain their exponential growth phase. They were collected in culture medium after dislodging from the flask substrate with a cell scraper. Cells were incubated with 1 *μ*M calcein-AM green (Life technologies) for 30 min at 37 °C for fluorescent labeling.

### Immunomagnetic labeling

2.5 *μ*l streptavidin-coated superparamagnetic polystyrene beads (4 × 10^8^ beads/mL, 4.5 *μ*m diameter, Dynabeads, Life technologies) were conjugated with 0.25 *μ*g biotinylated anti-CD11b antibody (Clone M1/70.15, Life technologies) by incubating in 1 ml PBS overnight at 4 °C. The tube containing antibody conjugated magnetic beads was then placed in magnet (Dynamag-5, Life technologies) for 1 min and discard the supernatant to remove excess of antibodies. 1 ml fluorescently labeled RAW 264.7 cells in PBS were added to the beads (cell number: bead number = 1:4) and mixed well by pipetting. The mixture was incubated for 1 h at 4 °C on a rotator mixer at 24 rpm. Cell-bead complexes were then centrifuged and resuspended in PBS containing 7 wt% dextran (Sigma-Aldrich). Dextran was used here to increase density of PBS to prevent cells from settling in the tubing or channel^38^. After the procedure, cells were put into 96 well-plates to determine the percentage of cells being magnetically labeled by visual inspection under a microscope.

### Magnetic structure fabrication

The process is shown in Fig. 1a. A microfluidic mold was fabricated first. Briefly, a photomask with microscale pattern was designed by FreeHand MX software (Macromedia) and printed at a resolution of 5,080 dpi on transparencies (Infinity Graphics). The features on the mask were transferred to a silicon wafer spin-coated with 10 *μ*m thick photoresist AZ 9296 (Clariant) after UV exposure and development. A 10:1 mixture of polydimethylsiloxane (PDMS, General Electric Silicones RTV 615, MG chemicals) prepolymer was poured onto the silicon wafer to form a ~5 mm thick layer and cured in an 80 °C oven for 1 h, followed by being peeled and drilled for inlet and outlet. The PDMS structure was washed with liquid soap (Thermo Scientific), sterilized with 70% ethanol, rinsed with ultrapure water, and blow-dried with air before being put in contact with a pre-cleaned glass slide (1 mm thick) for bonding by reversible surface adhesion. The chip (including the glass slide and the PDMS structure) was baked at 80 °C for 30 min to strengthen the bonding. Ferrofluid (MJ300, Liquid Research, United Kingdom) was added to a reservoir of the chip and entered the channel by gravity. The chip was put under house vacuum for 30 min to facilitate filling of ferrofluid into the channel. The ferrofluid-filled chip was baked for 15 min each at 30, 40, 50, and 60 °C sequentially, followed by an overnight baking at 70 °C. The PDMS structure was then peeled off from the glass slide (which bears the magnetic structure). The glass surface was cleaned carefully with acetone-dipped cotton stick to leave magnetic stripes.

### Microfluidic chip fabrication

The microfluidic channel was fabricated by standard soft lithography^45^,^46^. Negative photoresist SU8 2075 (Microchem) was used to prepare a master for a channel with a depth of 100 *μ*m. After PDMS molding and drilling for inlet and outlet holes, the PDMS replica was aligned and bonded to the glass bearing the magnetic structure after oxidization of both surfaces in a plasma cleaner (Harrick Plasma). The assembled chip was baked at 80 °C for 1 h to ensure strong and irreversible bonding between PDMS and the glass.

### Microfluidic chip operation

The experimental setup used for microfluidic immunomagnetic separation is shown in Fig. 2b. The microfluidic channel was pretreated with 1% BSA in PBS for 1h at 37 °C to minimize nonspecific adhesion of cells and beads to the channel walls. Microfluidic chip was set up with the glass surface facing up and a NdFeB magnet with maximum energy product of 42 MGOe, (1/4″ × 1/4″ × 1/4″, K&J Magnetics) was placed on the glass slide. The magnetization direction of the permanent magnet is perpendicular to the flow and the channel width. This setup ensured that the distance between the magnet and the channel was consistent among experiments (~1 mm). With the magnet out of the observation path, this also allowed real-time monitoring of cells within the channel with an inverted fluorescent microscope. RAW cells conjugated with magnetic microbeads were flown through the microfluidic channel at a constant rate sustained by a syringe pump (Harvard Apparatus).

### Quantification of cell capture

Cells suspended in PBS with 7 wt% dextran were counted with a hemocytometer and diluted to be roughly 10 cells/*μ*l before being pumped into the channel. The accurate cell concentration was determined by counting cells in 20 *μ*l suspension with a 96-well plate. Cell capture efficiency was defined as in equation (1):


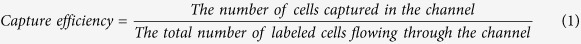


when the flow velocity was higher than 10 mm/s, the total number of magnetically labeled cells entered was determined by the percentage of labeled cells (89% for RAW 264.7), the cell concentration, and the volume of solution being pumped through the chip. The captured cells in the channel were imaged and counted in ImageJ software. Inverted fluorescence microscope (IX-83, Olympus, NY) equipped with a 2X dry objective, a UV lamp and a CCD camera (Hamamatsu, NJ) was used for imaging. The excitation light was filtered by a D480/40 filter, and the emission light was filter by a D535/40 filter. When the flow velocity was lower than 10 mm/s, cell density was not uniform during the experimental duration, thus we determined the number of labeled cells by imaging. Videos were taken at rate of 6 frames per second at the entrance and exit of the channel and the number of labeled cells during a time interval was counted.

### COMSOL Modeling

3D steady-state model was built in COMSOL Multiphysics 4.3 to simulate the distribution of magnetic field within the microfluidic channel. We assumed no free current in the region and selected the Magnetic Field, No Currents interface from the AC/DC Module for the modeling. Instead of using infinite element domain, a sufficiently large air box (20 mm × 20 mm × 20 mm) was draw to cover the whole magnetic block and magnetic structures. In a current free region,


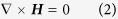


Scalar magnetic potential, *ψ*, is defined as:


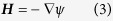


For the paramagnetic stripes and surrounding air:


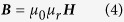


and for the permanent magnet:


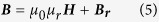


where *μ*_0_ is the permeability constant, *μ*_r_ is the relative permeability determined by the materials, and ***B***_***r***_ is the residual flux density of the permanent magnet (1.3 T). *μ*_r_ was estimated to be 200 for the magnetic stripes and 1 for air and the permanent magnet.

Together with the Guass’s law:


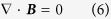


*ψ* can be solved. Magnetic insulation condition was used as the boundary condition. Initial value of *ψ* is set to be zero.

To model the magnetic field gradient *d**B***/*dy* and *d**B***/*dx*, fine mesh (maximum element size of 2.5 *μ*m and minimum element size of 0.25 *μ*m along y/z axis; 20 times larger mesh size along x axis) was used for the region covering the three magnetic stripes at the center of the 7-stripe-structure. One additional air box of 4 mm (W) × 0.5 mm (D) × 6 mm (L) was added to enclose all 7 stripes with a coarse mesh (max. 20 *μ*m and min. 2 *μ*m along y/z axis). This mesh size was also applied to the 4 magnetic stripes other than the 3 central ones. Large mesh size (max. 400 *μ*m and min. 4 *μ*m along y/z axis) was used for the permanent magnet and the outer large air box. Cubic discretization was used for high precision.

## Additional Information

**How to cite this article**: Sun, C. *et al*. Paramagnetic Structures within a Microfluidic Channel for Enhanced Immunomagnetic Isolation and Surface Patterning of Cells. *Sci. Rep.* **6**, 29407; doi: 10.1038/srep29407 (2016).

## Supplementary Material

Supplementary Information

Supplementary Movie S1

Supplementary Movie S2

Supplementary Movie S3
