# Supplementary material for: Paramagnetic Structures within a Microfluidic Channel for Enhanced Immunomagnetic Isolation and Surface Patterning of Cells
Source: Sci Rep. 2016 Jul 8;6:29407. doi: 10.1038/srep29407 (PMC4937384; doi:10.1038/srep29407)
Supplement: Supplementary Information [file srep29407-s1.pdf]

Paramagnetic Structures within a Microfluidic Channel for Enhanced Immunomagnetic Isolation and Surface Patterning of Cells

Chen Sun, Hamid Hassanisaber, Richard Yu, Sai Ma, Scott S. Verbridge, Chang Lu*

Email: changlu@vt.edu

Supplementary Information

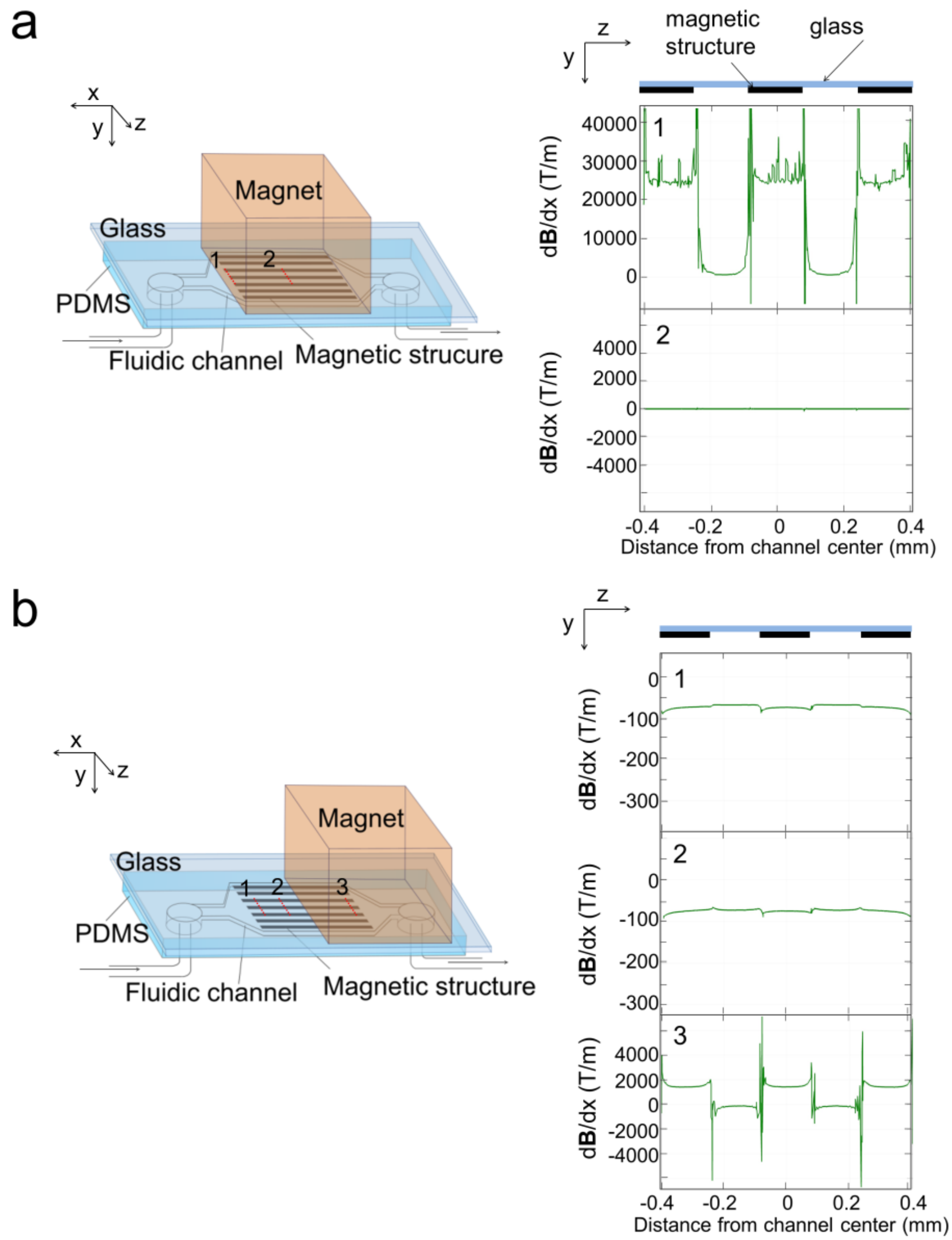

Figure S1. COMSOL modeling of ***dB****/dx*. (a) COMSOL modeling of ***dB****/dx* (in the plane that is within the channel and 10 µm from the glass surface) when the magnet is placed right above, covering the magnetic structure. ***dB****/dx* was plotted along the channel width at various locations (1 and 2) along the channel length. (b) COMSOL modeling of ***dB****/dx* when the magnet covers only half of the magnetic structure. ***dB****/dx* was plotted along the channel width at various locations (1, 2, and 3) along the channel length. The dimensions of the microfluidic channel were 2.4 mm (W, z direction) × 100 *µ*m (D, y direction) × 10 mm (L, x direction), and the magnetic structure contained 7 stripes of 160 *µ*m (W, z direction) × 4.5*µ*m (D, y direction) × 5 mm (L, x direction) uniformly aligned in the channel.

The supplementary movies can be played by QuickTime or Windows Media Player.

In all these movies, cells were flowed into the microfluidic channel at 1 mm/s. The dimensions of the microfluidic channel were 2.4 mm (W) ×100 μm (D) × 10 mm (L), and the magnetic structure contained 7 stripes of 160 μm (W) × 4.5 μm (D) × 5 mm (L), aligned in the direction of the flow. Cells were fluorescently labeled with calcein AM green. The movie was captured at a speed of one frame every 5s and played at 5 fps.

Movie S1. The movie shows the movement of magnetically labeled cells when they flow through the microfluidic channel with setting showed in Fig. 4a (when the magnet covers the entire magnetic structure from above).

Movie S2. The movie shows the movement of magnetically labeled cells when they flow through the microfluidic channel with setting showed in Fig. 4c (when the magnet covers only half of the magnetic structure from above).

Movie S3. The movie shows the movement of magnetically labeled cells when they flow through the microfluidic channel without magnetic stripes.
